# Asymptomatic Lymphocytic Interstitial Pneumonia with Extensive HRCT Changes Preceding Sjogren's Syndrome

**DOI:** 10.1155/2021/6693031

**Published:** 2021-01-07

**Authors:** Hazlyna Baharuddin, Mohammad Hanafiah, Syazatul Syakirin Sirol Aflah, Mohd Arif Mohd Zim, Shereen Suyin Ch'Ng

**Affiliations:** ^1^Faculty of Medicine, Universiti Teknologi MARA, Malaysia; ^2^Hospital Selayang, Malaysia; ^3^Assunta Hospital, Malaysia; ^4^Institut Perubatan Respiratori, Malaysia

## Abstract

Lymphocytic interstitial pneumonia (LIP) is a rare condition, commonly associated with Sjogren's syndrome (SS). We report a 53-year-old woman with an incidental finding of an abnormal chest radiograph. LIP was diagnosed based on high-resolution computed tomography and lung biopsy, but treatment was not initiated. Six years later, she developed cough and dyspnoea, associated with dry eyes, dry mouth, and arthralgia. While being investigated for the respiratory symptoms, she developed cutaneous vasculitis and was treated with 1 mg/kg prednisolone, which resulted in the improvement of her respiratory symptoms. Physical examination revealed fine bibasal crepitations, active vasculitic skin lesions, and a positive Schirmer's test. Investigations revealed a restrictive pattern in the pulmonary function test, stable LIP pattern in HRCT, and positive anti-Ro antibodies. She was treated with prednisolone and azathioprine for 18 months, and within this time, she was hospitalised for flare of LIP, as well as respiratory tract infection on three occasions. During the third flare, when she also developed cutaneous vasculitis, she agreed for prednisolone but refused other second-line agents. To date, she remained well with the maintenance of prednisolone 2.5 mg monotherapy for more than one year. The lessons from this case are (i) patients with LIP can be asymptomatic, (ii) LIP can precede symptoms of SS, and (iii) treatment decision for asymptomatic patients with abnormal imaging or patients with mild severity should be weighed between the risk of immunosuppression and risk of active disease.

## 1. Introduction

Lymphocytic interstitial pneumonia (LIP) is a rare condition. Cha et al. reported that among 1,167 lung biopsies of patients with interstitial lung disease (ILD) collected over 14 years, only 15 were found to have LIP [1]. LIP is regarded as both a disease (rare idiopathic interstitial pneumonia) and as a nonneoplastic, inflammatory pulmonary reaction to various external stimuli or systemic disease [1]. It is commonly found in connective tissue disease (CTD) especially Sjogren's syndrome (SS). A systematic review of pulmonary involvement in SS reported that of 146 histopathological diagnoses, the most common was nonspecific interstitial pneumonia (45%), followed by bronchiolitis (25%), usual interstitial pneumonia (16%), and lymphocytic interstitial pneumonia (15%) [2]. We report a case of asymptomatic LIP with extensive HRCT changes before she became symptomatic and developed features of SS, six years later.

## 2. Case Presentation

**Figure 1:**
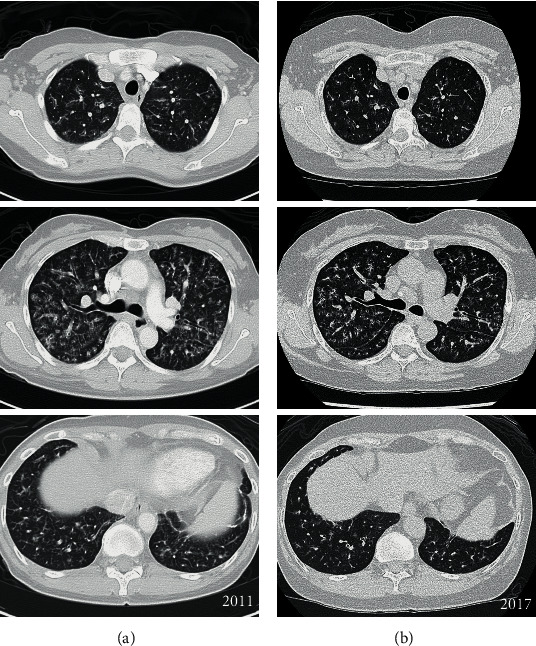
Selected axial CT images of the initial scan (a) and 6 years later (b) of LIP. There are diffuse peribronchovascular thickening and ground glass changes with tiny cysts along the bronchovascular bundles in both lungs.

A 53-year-old woman was incidentally found to have an abnormal chest radiograph during preoperative assessment for elective hysterectomy, ten years ago. She sought treatment in a hospital in a neighbouring country and was diagnosed with LIP, based on high-resolution computed tomography (HRCT) finding (Figure 1(a)) and lung biopsy report of marked fibrosis of interlobular septa, diffuse interstitial and peribronchiolar lymphoplasmacytic infiltration with scattered eosinophils. She did not receive any treatment and subsequently defaulted follow-up after two years.

Six years after the incidental abnormal chest radiograph, she consulted a respiratory physician with an eight-month history of cough and dyspnoea. Six-minute walk distance was 440 m with oxygen saturation of 89%, and pulmonary function test (PFT) showed a restrictive pattern with FEV1 53%, FVC 67%, and DLCO 70%. A repeat HRCT showed stable appearances of diffuse peribronchovascular thickening and ground glass changes with tiny cysts along the bronchovascular bundles in both lungs (Figure 1(b)). She was referred for a rheumatology consult a few months later when immunology investigations revealed positive antinuclear and anti-Ro antibodies. An additional history of dry eyes, dry mouth, and inflammatory arthralgia, which started three months after the onset of respiratory symptoms, was revealed. She also reported an improvement in her respiratory symptoms a month earlier, after she was prescribed prednisolone 1 mg/kg by a dermatologist for cutaneous vasculitis. At the time of the rheumatology clinic appointment, she was on prednisolone 20 mg and she reported recurrence of cutaneous vasculitis. Physical examination revealed a 52 kg lady with oxygen saturation of 93% on room air at rest, fine crepitations at the lung bases, and multiple vasculitic lesions on the lower limbs. Schirmer's test was positive. Abnormal blood investigations included anaemia (haemoglobin 10.8 g/dL), thrombocytosis (platelet 494 × 10^9^/L), raised erythrocyte sedimentation rate (86 mm/hour), and hypergammaglobulinaemia (globulin 63 g/L). She also had pulmonary hypertension (pH), confirmed by right heart catheterisation showing mean pulmonary arterial pressure of 26 mmHg and precapillary wedge pressure of 9 mmHg.

LIP secondary to primary SS was diagnosed. Prednisolone dose was increased to 0.75 mg/kg and azathioprine 2 mg/kg was started as a steroid-sparing agent. Within the next 18 months, she had three hospitalisations due to worsening respiratory symptoms. She was treated for ILD exacerbation (with high-dose prednisolone) and respiratory tract infection (with intravenous antibiotics). During her last flare, she also developed a recurrence of cutaneous vasculitis. At this time, she agreed for prednisolone but refused steroid-sparing agent. Surprisingly, she remained stable and for the last 10 months; she was well on prednisolone 2.5 mg. She was content with the status quo of NYHA Class II and regular four-monthly clinic follow-up, without any hospitalisations.

## 3. Discussion

The disease course in our patient highlights some important lessons. First, LIP is a rare disease, and secondary causes should be sought. In some patients, pulmonary involvement precedes other systemic symptoms of CTD, making the distinction between idiopathic ILD and CTD-ILD impossible at the time of diagnosis [3]. Nonetheless, the involvement of multidisciplinary teams is advocated to ensure optimal management of ILD. The diagnosis of SS was delayed in our patient because her rheumatologic symptoms (inflammatory arthralgia, dry eyes, and dry mouth) were not explored when she first presented with cough and dyspnoea. In fact, they were only discovered months later by the rheumatologist, who was referred for positive ANA and anti-Ro antibodies. Without the presence of rheumatologic symptoms and signs, she would be classified as Interstitial Pneumonia with Autoimmune Features (IPAF) [4], as she fulfilled a serologic and morphologic domain only. Some may argue that the classification of IPAF or CTD-ILD is not important as the treatment for both conditions is the same. However, patient monitoring in CTD is different because of multisystem involvement.

Secondly, our patient remained asymptomatic for six years despite extensive HRCT changes. Although CT is the most sensitive method of detecting lung abnormalities, radiological abnormalities do not correlate with pulmonary function tests and respiratory symptoms [5, 6]. The respiratory manifestation of SS is polymorphic and varies in severity [5]. The severity of pulmonary involvement is graded according to PFT results and functional class of patients with HRCT-proven ILD [7]. It is classified as low activity in patients with chronic respiratory symptoms associated with mucosal dryness of the upper respiratory tract with normal imaging and in asymptomatic patients with altered pulmonary imaging [7]. Our patient's disease activity was low in the first six years because she was asymptomatic, but later became moderate because of abnormal PFT and NYHA Class II.

The final lesson is to appreciate that not all pulmonary manifestations in SS need treatment especially those who are well with stable disease [5]. Although there is no conclusive standard therapy for SS with pulmonary involvement, immune therapy such as corticosteroid and/or immunosuppressive drugs is indicated when there is a presence of progressive chest symptoms, impaired respiratory function, or prominent abnormal chest or HRCT [5]. In cases such as our patient, treatment decision with cytotoxic drugs should be weighed carefully between the risk of rendering patients immunocompromised and the risk of patients developing active disease. Patients with LIP generally respond well to initial corticosteroid therapy, but up to one-third may die within several years of diagnosis from the progression of the disease or infectious complications related to immunosuppressive therapy [8]. Based on our experience, we can conclude that LIP in our patient is steroid-responsive, and steroid-sparing agent did not alter her disease course, as she had been stable on a low dose of prednisolone for more than a year.
